# The 4Ds of Dealing With Distress – Distract, Dilute, Develop, and Discover: An Ultra-Brief Intervention for Occupational and Academic Stress

**DOI:** 10.3389/fpsyg.2020.611156

**Published:** 2020-12-16

**Authors:** Warren Mansell, Rebecca Urmson, Louise Mansell

**Affiliations:** ^1^CeNTrUM (Centre for New Treatments and Understanding in Mental Health), Manchester Academic Health Science Centre, Division of Psychology and Mental Health, Faculty of Biology, Medicine and Health, School of Health Sciences, University of Manchester, Manchester, United Kingdom; ^2^Beyond Psychology, Rochdale, United Kingdom

**Keywords:** resilience, stress management, online interventions, problem-solving, single session therapy, brief therapy, emotion regulation, expressive writing

## Abstract

The Covid-19 crisis has clarified the demand for an ultra-brief single-session, online, theory-led, empirically supported, psychological intervention for managing stress and improving well-being, especially for people within organizational settings. We designed and delivered “4Ds for Dealing with Distress” during the crisis to address this need. 4Ds unifies a spectrum of familiar emotion regulation strategies, resilience exercises, and problem-solving approaches using perceptual control theory and distils them into a simple four-component rubric (Distract–Dilute–Develop–Discover). In essence, the aim is to reduce distress and restore wellbeing, both in the present moment through current actions (distract or dilute), and through expressing longer-term goal conflicts (e.g., through talking, writing, and drawing) to discover new perspectives that arise spontaneously after sufficient time and consideration. The intervention is user-led in that it draws on users’ own idiosyncratic and pre-existing experiences, knowledge, skills and resources to help them apply an approach, or combination of approaches, that are proportionate and timed to the nature and context of the stress they are experiencing. In this article we review the empirical basis of the approach within experimental, social, biological and clinical psychology, illustrate the novel and time-efficient delivery format, describe its relevance to sports and exercise, summarise feedback from the recipients of the intervention to date, and describe the directions for future evaluation.

## Scientific Background

The Covid-19 crisis has brought the mental health needs of the population into greater awareness. In particular, the effects of lockdown on employment, interpersonal relationships, access to nature, and the provision of leisure activities and exercise has reminded those in government, academia and professional practice of the important role that these factors play in mental health and well-being (Kumar and Nayar, 2020). When they are unavailable, compromised or less accessible, wellbeing and mental health are also compromised (Molarius et al., 2009). This has prompted active discussion of how to replace their buffering effects on stress within the general population, and within organizational settings such as schools, universities, sports organizations and businesses (Maben and Bridges, 2020).

There are a wide variety of programs provided in workplaces that are aimed at helping people deal with distress. The effectiveness of these interventions is well established, but mainly in the short term (Lamontagne et al., 2007). To promote access and scalability, many stress management interventions are delivered online or via computer, and vary in length from 2 to 12 weeks of modules (Heber et al., 2017). They may include training sessions grounded in cognitive behavioral therapy, mindfulness training, career planning, or personal resilience training, and even brief (under 4 weeks) interventions show small-but-significant reductions in stress (Heber et al., 2017). However, most interventions are atheoretical or eclectic in nature, and delivered over at least 3 weeks (Ryan et al., 2017). The scientific and professional background has largely recommended intensive training of specific techniques for specific issues (Yusufov et al., 2019).

Even the interventions proposed to be theory-driven appear to be more eclectic in nature and involve intensive training. For example, the role of control in dealing with stress formed the basis of an online intervention in college students (Hintz et al., 2014). This intervention was found to be superior to a stress-information-only comparison group in its effects on depression, anxiety and stress at post-intervention and at 3-week follow-up. The intervention was fairly intensive, involving four modules over 2 weeks, and including between-session homework exercises and stress logs. It amalgamated the theory of present control (Frazier et al., 2001) with self-efficacy theory (Bandura, 1986) and questioning drawn from motivational interviewing (Rubak et al., 2005).

From the above brief synthesis of the literature, we conclude that there is distinct niche for an approach that is guided by a single theory, and is ultra-brief yet focused on facilitating long-term change. We have devised an approach of this kind, termed “The 4Ds of Dealing with Distress.” In order to do so, we reviewed the research evidence regarding the components of existing interventions to identify which classes of strategies appear to be effective in reducing distress both in the short and long term. We then produced an integrative framework for these components united by a single theoretical framework – perceptual control theory (PCT) – although the theory is not explicitly “taught” in the session. In the current article we draw out the particular relevance for our intervention to the psychology of human performance, exercise, sports and the wellbeing of clients and staff within sports organizations.

## Why Perceptual Control Theory?

There are several reasons for utilizing PCT. First, PCT is grounded within physiology, physics and engineering, which can make it directly applicable to a number of professions, including sports professions. For example, PCT as been applied to movement science, including hand-eye coordination (Powers, 1999), catching flyballs (Marken, 2005), intercepting objects such as frisbees (Shaffer et al., 2004), and dual agent pursuit (Sandhu et al., 2020). Second, PCT is interdisciplinary, and applied widely throughout the fields of mental health interventions (Alsawy et al., 2014), organizational psychology (Vancouver, 2020), human-computer interaction (Farrell et al., 1999), and education (Zhao and Cziko, 2001). Thus, it provides a direct link between psychology, technology, business, and education – the domains traversed by an online wellbeing intervention for occupational and educational organizations.

According to PCT, control is at the heart of adaptive functioning, and so control is the essence of health, wellbeing and mental health (Powers et al., 1960; Carey et al., 2014; Carey, 2016). Within PCT, distress is the loss of control that emerges when a person cannot achieve and maintain the experiences that are most important to them. There is substantial evidence from experimental research to support such a claim (see Table 1, Control). In relation to this core tenet of PCT, the third reason for utilizing PCT is that several sources of evidence point to the benefits of acknowledging and embracing client control, choice and user-tailored interventions. It is well established that individuals vary widely in their readiness and pace of change (Hayes et al., 2007). There is also evidence that having control over stressful experiences, such as facing a fear, is beneficial (Healey et al., 2017). When users are interviewed about how they utilized existing interventions, they commonly report selecting and personalizing the strategies and insights that they find relevant to their own specific situation (Amos et al., 2019; Warwick et al., 2019). Building on these features, when clients are given the control to book their own timing and duration of appointments, the benefits include a reduced waiting list (Carey and Spratt, 2009) and a greater efficiency of the intervention relative to benchmarked studies (Carey et al., 2013). A previously developed group intervention based on PCT – known as the Take Control Course (TCC) – has an emerging evidence base (Morris et al., 2016). This supports the principles of a psychological intervention based on PCT; however, unlike 4Ds, the TCC is six weekly sessions, provides more in-depth explanation of theoretical principles, and was initially designed for people within mental health services to access in person rather than online.

**TABLE 1:** The components of 4Ds, their definition, examples, and evidence.

Component	4D definition	Examples	Selected evidence from experimental research	Selected evidence regarding neural pathways
Control (an underpinning theoretical construct of 4Ds)	The user chooses, achieves and maintains their desired experiences.	Draw on users’ knowledge, skills, and experiences. Encourage users to consider, select, and test each strategy for themselves.	Loss of control is associated with distress and control is associated with wellbeing (Cheng et al., 2013; Tabibnia, 2020).	Chronic loss of control through dorsal raphe nucleus of the brain, reversal via medial prefrontal cortex (Maier and Seligman, 2016).
Distract	Everyday activities that shift attention away from distress for a period of time and improve mood.	Sport, exercise, social activities, nature engagement, music, daydreaming, and humor.	Distraction improves short-term mood (Webb et al., 2012); rewarding, and social, activities reduce distress (Tabibnia, 2020).	Multiple combined pathways including reward pathways (e.g., ventral striatum) (Tabibnia, 2020).
Dilute	Trained techniques that reduce current distress.	Applied relaxation, slow breathing, grounding, brief mindfulness, and thought challenging.	Applied relaxation is effective for anxiety (Kim and Kim, 2018); mindfulness training reduces stress (Khoury et al., 2015).	Reduced activation of autonomic nervous system and release of cortisol (Pascoe et al., 2017).
Develop	Plans to engage in discovery in a safe and constructive way.	Worry time, self-scheduling, drawing upon times they have previously coped and generating compassionate imagery.	Control over worry linked to reduced distress (LaFreniere and Newman, 2019); self-compassion interventions reduce distress (Ferrari et al., 2019).	Compassion recruits midbrain periaqueductal gray (PAG) (Simon-Thomas et al., 2012).
Discover	Shifting to and sustaining engagement with, and expressing the experience of distress to explore the source of conflicts to generate new perspectives.	Emotional disclosure, discovery talk (active listening), and expressive writing or drawing.	Active listening reduces distress (Jones and Cutliffe, 2009); expressive writing improves wellbeing (Travagin et al., 2015); effects of expressive writing mediated by conflict awareness (Kelly et al., 2012); higher level construals enhance personal growth (Wang et al., 2016).	Medial prefrontal cortex, amygdala, and autonomic nervous system (Saunders et al., 2017; Tabibnia, 2020).

A fourth reason for utilizing PCT lies in its focus on core principles, rather than specific techniques, tools or strategies. This allows PCT to be applied from a “meta” perspective to an array of pre-existing methods available to each individual. So, as will be described later, an array of expressive methods – such as expressive writing (Kelly et al., 2012) and artistic expression (Stevenson-Taylor and Mansell, 2012) – can facilitate the mechanism of change described by PCT.

Table 1 summarizes the components of 4Ds with examples and evidence.

## Theoretical Rationale for 4Ds

In everyday life, loss of control manifests itself as uncertainty, worry, low mood, intrusive memories, poor motivation, and various other “stress symptoms.” Therefore, any opportunity to act in a way that restores control over these experiences will relieve distress in the short term – we have termed these “distraction” and “dilution.” Examples of distraction are hobbies and other pleasurable activities, as well as internal processes such as daydreaming. Distraction is a commonly used term to describe common, everyday activities that intentionally shift attention away from one’s problematic thoughts and feelings for a period of time and may even improve mood (see Table 1). Some of these activities, such as sport, exercise, and nature engagement, whilst being “distracting,” may tap into other more significant pathways to well-being that overlap with the later components of 4Ds, such as providing opportunities to safely reflect on problems, or to become aware of higher level values, such as health during exercise and connectedness with others during team sports (Hoye et al., 2012). We save the term “dilution” for those novel, specialist-trained activities that aim to reduce heightened states of distress in the moment, and they include mindfulness training, slow breathing techniques, grounding methods, and applied relaxation.

Both distraction and dilution may restore control very effectively in the short term but at the same time not resolve long-term issues, or even have the potential for generating other problems. For example, distracting oneself with taking on extra work can help a person feel that they are meeting their standards for productivity, but failing to meet their standards for spending time with the family. There is even evidence that inflexible engagement with sports or exercise have “addictive” properties (Bamber et al., 2000; Nogueira et al., 2018). Engaging in mindfulness mediation for 45 min every day may provide a sense of control over negative thoughts as one decenters from them, but take one’s attention away from thoughts about pressing issues (paying fines, housing repairs, and dealing with threats to employment) that need to be addressed as soon as possible. Thus, the PCT approach encourages each individual to work out the most adaptive and flexible use of any approach rather than providing any blanket advice about specific strategies. There is considerable evidence from systematic reviews that psychological flexibility is closely associated with mental health and wellbeing (Kashdan and Rottenberg, 2010; Morris and Mansell, 2018), and a PCT approach to the use of behavioral or cognitive strategies proposes that clients are provided with the opportunity to weigh up the pros and cons of these as applied to their own life context (Alsawy et al., 2014).

From a PCT perspective, the long-lasting source of distress is not a lack of resource, strategy or skill, but an underlying conflict between high-level goals (e.g., ideals, standards, and principles) of similarly high importance. There are a variety of examples of goal conflict occuring in the context of sports and exercise (Graham and Dixon, 2017). For example, an experimental study of students who had the goal of exercising found that priming the conflicting goal of performing well academically led to evidence of reduced motivation to engage in physical exercise (Bailis et al., 2011). The range of potential goal conflicts are highly idiosyncratic. For example, a manager of a gym may want to maintain their sense as a trustworthy person by re-employing all of her staff after lockdown, but at the same time want to keep her family financially secure through streamlining staffing at the organization. Taking another example, an employee experiencing bullying from a colleague may be in conflict between seeking help to stop the bullying, and keeping the experience private for fear of being ridiculed or experiencing further retaliation.

According to PCT, goal conflict is commonplace and it is normally resolved when a conflict is brought into awareness through speech, or through other forms, such as writing or drawing. Therefore, distress reduction is ultimately a process of “discovery” through expression, feedback of one’s expressed ideas, and the curiosity that helps guide awareness to the source of the conflict. Because the process of change is spontaneous – sometimes getting worse before it gets better – it can feel destabilizing in itself. The individual therefore needs to be in control of the discovery process, even if it requires the help of another person such as a curious listener. It is this further user-led element of the 4Ds that is termed “develop” and prompts the user to plan and prepare the most suitable conditions to set forth into, the sometimes open-ended, discovery process.

The role of the four components can be described with an example. The staff member experiencing bullying may:

(a) Regain short-term control outside the workplace by effectively *distracting* themselves from their feelings of failure through focusing on their productive work from home, sporting activities, and their rewarding family life.
(b) Regain short-term control in the work place by *diluting* their feelings of panic when at work through breaking the cycle of escalating arousal with slow breathing and applied relaxation.
(c) Regain long-term control by *developing* opportunities to express their experiences of bullying. This begins by starting a private expressive writing journal, and shifts to open conversations with their partner, during which childhood experiences of bullying are also described and similar conflicts explored. Among other insights, this staff member *discovers* that their overall goal of being respected and valued could be achieved by describing the details of the bullying incidents privately to a manager who has the same qualities as a considerate teacher who helped them as a child.

In summary, distress is the loss of control over valued life experiences. The loss of control may persist or resolve to various degrees at various timescales, depending on the individuals and the nature of the problem. In some instances, activities that distract from, or dilute, the distress can be enough to regain enough short-term control whilst the source of distress resides, or whilst it is resolved naturally through pre-learned attempts at discovery that shift and sustain awareness to the source of the confict (e.g., talking through one’s mixed feelings with an untrained friend). In many instances, however, the individual will need to plan and prepare (develop) a more regular format to engage in extended discovery, such as doing regular expressive writing, or booking a time to talk to a person who is trained to help them shift and sustain their awareness on the source of the problem.

## Delivery

**FIGURE 1:**
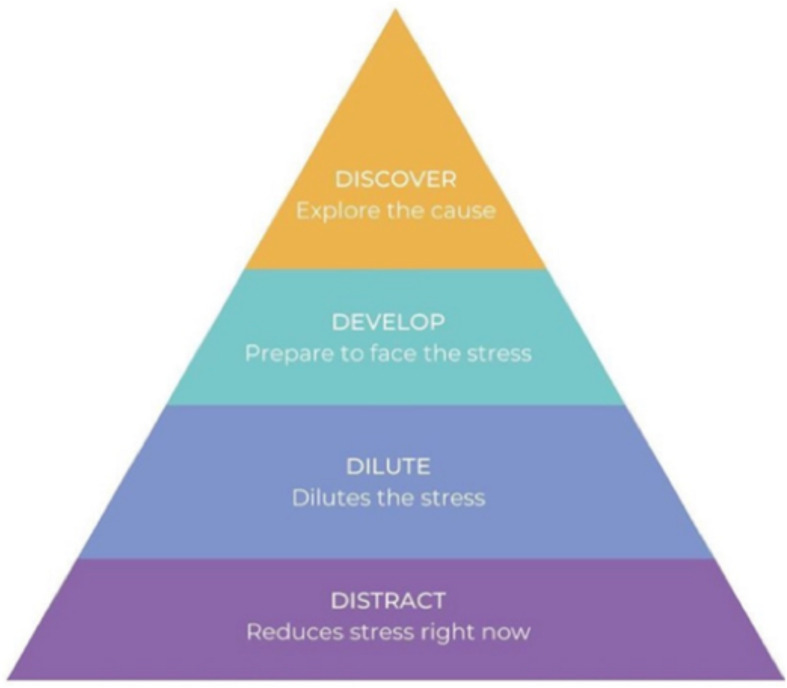
The 4D pyramid.

4Ds integrates four components that are organized as levels of a pyramid (see Figure 1). The idea is to work up the pyramid during the one 60–90 min session that explains the approach. The session points out that all four Ds (Distract, Dilute, Develop, and Discover) can be helpful, but that only the person experiencing distress can work out which balance of the 4Ds will address the problems at the root of their current distress. Depending on the source and intensity of stress, some problems may resolve with simple approaches at the bottom of the pyramid, whereas others may require working upward, entailing full use of the “higher” levels of the intervention.

Clients are encouraged to draw on their own strengths, activities, preferences and resources, throughout the session right from the beginning. What works for one person with one problem may not work for a different problem, or for a different person. Given the theoretical emphasis on control, diversity, flexibility and self-determination are encouraged over and above intensive training in a specific method. In fact, the aim of 4Ds is to enable each user to integrate the methods they have already encountered and started to use, rather than to train intensively in any new techniques. For many users who have experienced training in all kinds of well-being techniques to date, this user-led emphasis can be refreshing, or even pivotal in their appreciation of the 4Ds approach. 4Ds is focused on explaining *how* and *why* wellbeing approaches may work so that the individual can find their own, unique combination of solutions.

Prior to the session, clients are informed of the experiential aspect of trying the techniques during the session and are encouraged to think about current stressors that they would like to explore throughout the session. Furthermore, clients are provided with a worksheet to complete during the session, detailing which strategies work for them, in each of the four components. The worksheet also serves as a reminder that they have choice over how to deal with the stress and therefore they have control (worksheets available from authors).

The session describes explicit parallels between the 4Ds and the range of approaches one takes to any kind of problem – this is typically grounded in the expertise of the individual clients within the session. For example, clients with a health or exercise background are reminded of how they might deal with the physical pain from an injury. In the very short term – distraction may help – for example for a football player to get through a match by focusing on the game if they can manage it. Typically though, some form of dilution is also necessary – an analgesic to reduce the pain, for example. Yet, we are all aware that a significant injury will require more active exploration. The person with the injury will plan to consult a physiotherapist, or a doctor, who will do various tests and potentially scans, to attempt to discover which parts of the body are injured, as this will inform the appropriate intervention. Thus, there is a stage that involves development of a plan to make the appointment and preparing what to say to the health professional, and there is a stage that involves the actual exploration to discover the nature of the problem. The “catch” for problems with emotional distress is that only the person experiencing the distress can fully explore what is behind it. Only the symptoms, but not the source, can be identified by an expert on the outside with a test or a scan. A person experiencing distress is like a car looking under its own bonnet to work out what part is not working right.

The session then proceeds to describe the base of the pyramid – the client-led coping activities that act as a temporary distraction from distress. Their aim is to temporarily remove the experience of distress and clients often share forms of exercise and sporting activities as sources of distraction. Many of these activities also potentially serve important personal goals for the individual, thereby indirectly improving mood. For some people with some problems, distraction can be sufficient to redirect them away from their distress, so that they can find a way round the problems without extensively and intentionally exploring them. For example, during lockdown, one personal trainer lost her job at an indoor gym. She felt undervalued, sad and angry with her employer. One day she chose to distract herself from their feelings by running regularly with a local running club. At the club she met the manager of a personal trainer agency who offered her a well-paid role, which resolved her job stress in the short term. However, under different circumstances, distraction may purely allow the individual to mentally disengage from the stress for a brief period, offering only short-term relief and not tapping into the source of distress. In this case, users are encouraged to move up the pyramid to the second component.

At the next step up is a collection of very simple techniques (Dilute) that enable the client to circumvent some of the processes that temporarily exacerbate distress. Their aim is not to remove the experience of distress, but to reduce its intensity whilst still experiencing some, manageable, degree of distress. These techniques overlap with various techniques used in a variety of other approaches, but in 4Ds they are aligned under one principle; they provide the client with greater control at that moment. This may be control over physiological experiences (slow breathing and muscle tension), or thinking (mindful awareness and grounding techniques). Thus, the client gets a sense of control in the moment, and these techniques interfere with the actions that can perpetuate their current, most distressing states of mind.

In the third step of the session, the clients are introduced to the opposite side of the coin – the need to actually think about and talk about the problems they are having, even though this can feel distressing. The “Develop” aspect of 4Ds prompts them to be in control of this decision such that the pace, depth, timing and setting feels right for them, maximizing their ability to face the source of distress. Therefore, the concept of “worry time” is introduced – a specific time of the day or evening to think about their problems. They are also reminded to think of a time in their life that they were able to cope, to draw upon some of the same strengths and resources. They are also helped to form a compassionate image if they choose to – a template for the kind of person they would want to share their problems with, to hold in mind when expressing them.

The fourth step – “Discovery” proceeds from this point, now that the clients are aware of the means to control their engagement and exploration of their difficulties. The facilitators illustrate the benefits of speaking one’s problems out loud to hear them in one’s own voice. This begins in private, simply by muting the audio – “It’s OK to talk out loud to yourself – it doesn’t mean you are losing your mind!” In addition to wide accessibility and scalability, privacy of verbal expression is an additional advantage of the online format over and above a face-to-face group intervention. The clients are then encouraged to do the “upward arrow” approach, adopted from the Take Control Course (Morris et al., 2018). This is a succession of “What makes that important?” questions help them to become aware of the higher level goals, values, ideals, and principles that are challenged by their difficulties. They are also provided with an opportunity to use expressive writing and reflect on the experience of doing so (Pennebaker and Chung, 2011), and they are introduced to Discovery Talk (@DiscoveryTalk), a way of adapting Method of Levels therapy (Carey, 2006) for people who are not mental health professionals to use in their everyday settings. In doing so, they are directed toward simple ways of actively listening to one another to help explore a problem, without giving advice, interpretations, or solutions, and helped to notice the new perspectives that they gain from the experience (Churchman et al., 2019).

The session rounds off with a reminder that it is entirely up to each person whether, and how, they use the approaches that are covered – they are in control. A different combination will work for different people at different times, and that is as it should be. Worksheets for 4Ds and further guidance on its implementation are available from contacting the authors.

## Implementation and Feedback

To date, the 4D sessions have been delivered by qualified and trainee clinical psychologists via online platforms, namely Zoom and Microsoft Teams, to a variety of client groups. To date, sessions have been accessed by the general adult public, by teachers at Primary and Secondary schools, tutors at colleges, mental health professionals, and teenagers (aged 13–16); encompassing around 200 individuals of varying ages and backgrounds.

User feedback has been gathered verbally and via voluntary online surveys. Feedback suggests a reduction in immediate stress symptoms, increased awareness around the cause of stress, improved confidence in managing stress and a preference for the “develop” and “discover” components. The finding that clients tend to see the additional value in the “develop” and “discover” components is consistent with PCT, which specifies that awareness needs to shift and sustain toward higher level goals in order to facilitate longer term alleviation of distress. Additionally, the clients seem to appreciate that the approach is *universal*, in keeping with the universal principles of PCT, with clients expressing a keenness to try these with others too. For example, teachers spoke of the strategies being useful for their own stress but were also enthused to try them with pupils; indicative of the model being applicable to most ages and sources of stress. To date, suggestions for improvement typically involve the request for more opportunities to learn ways to engage in discovery, such a extended opportunities for expressive writing, or more training in the kinds of questions to ask to help another person explore their problems in more depth and detail. In response to this, we have included a follow-up session for some groups to extend the practice and to receive feedback on this last section of the course as well as referring them to wider resources such as online journaling and expressive writing resources, @DiscoveryTalk and videos of Method of Levels in action.

## Conclusion

We have summarized the scientific and pragmatic rationale for a novel, ultra-brief intervention for stress management that lends itself particularly well to the Covid-19 crisis, given the challenges to occupational, academic and family life, and the necessity for an easily accessed, one-session, remote format. Unlike alternative interventions, it entrusts clients with their own selection, use and testing of various strategies to meet the idiosyncratic and varying stressors in their own life. It builds upon techniques and principles that the clients will be familiar with but integrates them within one simple rubric and puts them in charge of how and when to use each approach. Whilst the science of the intervention is empirically supported, it requires systematic evaluation of its impact within this format, and of its putative mechanisms of action. Nonetheless, quantitative and qualitative audits have indicated that many clients grasp the key messages and report benefits. Given the challenging times we are in, it is imperative that practitioners and researchers work on the most efficient means to address stress and improve wellbeing, but in ways that enable expression and collective action rather than minimize and isolate suffering. The 4Ds is designed in such a way within its focus on fostering the agency of the individual to construct their own solutions, which may ultimately manifest at the individual, organizational, and societal levels.

## Data Availability Statement

The original contributions presented in the study are included in the article/supplementary material, further inquiries can be directed to the corresponding author/s.

## Author Contributions

WM and LM conceived of the clinical approach. WM led on the writing of the article and drawing upon the clinical experience of LM, WM, and RU in delivering the approach. All authors were involved in editing for the final manuscript.

## Conflict of Interest

The 4Ds is delivered by Beyond Psychology at a charge to users. LM is a director of Beyond Psychology, which is a not-for-profit social enterprise. The remaining authors declare that the research was conducted in the absence of any commercial or financial relationships that could be construed as a potential conflict of interest.
